# Bronchoscopy-Derived Correlates of Lung Injury following Inhalational Injuries: A Prospective Observational Study

**DOI:** 10.1371/journal.pone.0064250

**Published:** 2013-05-17

**Authors:** Samuel W. Jones, Haibo Zhou, Shiara M. Ortiz-Pujols, Robert Maile, Margaret Herbst, Benny L. Joyner Jr, Hongtao Zhang, Matthew Kesic, Ilona Jaspers, Kathleen A. Short, Anthony A. Meyer, David B. Peden, Bruce A. Cairns, Terry L. Noah

**Affiliations:** 1 Department of Surgery, The University of North Carolina at Chapel Hill, Chapel Hill, North Carolina, United States of America; 2 North Carolina Jaycees Burn Center, Chapel Hill, North Carolina, United States of America; 3 Department of Pediatrics, The University of North Carolina at Chapel Hill, Chapel Hill, North Carolina, United States of America; 4 Center for Environmental Medicine, Asthma and Lung Biology, The University of North Carolina at Chapel Hill, Chapel Hill, North Carolina, United States of America; 5 Department of Respiratory Care, University of North Carolina Hospitals, Chapel Hill, North Carolina, United States of America; 6 Department of Biostatistics, The University of North Carolina at Chapel Hill, Chapel Hill, North Carolina, United States of America; 7 Department of Anesthesiology, The University of North Carolina at Chapel Hill, Chapel Hill, North Carolina, United States of America; University of Leicester, United Kingdom

## Abstract

**Background:**

Acute lung injury (ALI) is a major factor determining morbidity following burns and inhalational injury. In experimental models, factors potentially contributing to ALI risk include inhalation of toxins directly causing cell damage; inflammation; and infection. However, few studies have been done in humans.

**Methods:**

We carried out a prospective observational study of patients admitted to the NC Jaycees Burn Center who were intubated and on mechanical ventilation for burns and suspected inhalational injury. Subjects were enrolled over an 8-month period and followed till discharge or death. Serial bronchial washings from clinically-indicated bronchoscopies were collected and analyzed for markers of cell injury and inflammation. These markers were compared with clinical markers of ALI.

**Results:**

Forty-three consecutive patients were studied, with a spectrum of burn and inhalation injury severity. Visible soot at initial bronchoscopy and gram negative bacteria in the lower respiratory tract were associated with ALI in univariate analyses. Subsequent multivariate analysis also controlled for % body surface area burns, infection, and inhalation severity. Elevated IL-10 and reduced IL-12p70 in bronchial washings were statistically significantly associated with ALI.

**Conclusions:**

Independently of several factors including initial inhalational injury severity, infection, and extent of surface burns, high early levels of IL-10 and low levels of IL-12p70 in the central airways are associated with ALI in patients intubated after acute burn/inhalation injury. Lower airway secretions can be collected serially in critically ill burn/inhalation injury patients and may yield important clues to specific pathophysiologic pathways.

## Introduction

Acute lung injury (ALI) and acute respiratory distress syndrome (ARDS) occur in a substantial proportion of patients following burns and inhalational injury, and are major factors associated with morbidity and mortality. Risk factors for lung injury include the inhalation of products of incomplete combustion containing a variety of toxins and particulate matter, direct cell damage, inflammation, infection, edema, and immunologic changes due to burn injury [1]–[3]. Animal models have identified multiple factors that contribute to lung injury and edema after toxic inhalations, including neuropeptide and bronchial artery blood flow [4]. Studies from our own research group suggest that surface burns affect systemic immunity and expression of toll-like receptors [5], [6]. However, few studies have attempted to explore pathophysiologic and host defense factors directly in humans.

Current therapy for inhalational injury and its pulmonary complications is supportive and focused on mechanical ventilation strategies, overcoming airway obstruction, and prevention or treatment of pneumonia [7], [8]. Serial bronchoscopies and airway washings for clearance of soot, debris and secretions are part of supportive care [9]–[11]. This clinical practice represents an opportunity to obtain lower airway secretions from patients for investigation of the relationships among mediators and clinical outcomes of interest.

We established a repository of bronchoscopic samples for analysis of airway mediators and their relationships with clinical outcomes. In an initial cohort of patients, we measured levels of selected mediators of inflammation, immunity, and tissue damage and repair. Our goal was to examine whether mediator profiles early after inhalational injury could be predictive of clinical outcomes, and if these observations could be used to develop discrete hypotheses regarding the mechanisms by which burn and inhalational injury modulate airway innate immunity. We here report data suggesting that specific airway cytokine patterns (increased IL-10 and decreased IL-12) in the first several days after burn/inhalational injury are associated with progression to lung injury.

## Materials and Methods

### Study Design

We carried out a single-center, prospective observational study of intubated patients with burns and/or suspected inhalation injury admitted to the North Carolina Jaycees Burn Center at the University of North Carolina Hospital at Chapel Hill, over an 8-month period. Patients were treated and underwent bronchoscopies under a standardized clinical protocol as described below. For study purposes patients were followed from the time of admission until discharge. All patients underwent at least one bronchoscopy soon after admission per clinical protocol. Since indications for subsequent bronchoscopies were determined by individual clinical factors, the total number and timing of bronchoscopies varied among patients studied.

For the research protocol, patients were included if they were intubated and mechanically ventilated for known or suspected inhalational injury. Clinical data including demographics, type of injury, and percent total body surface area burned were collected at admission. Additional clinical data including ventilator settings, appearance of the airway (see below), arterial blood gases, radiographic changes, and culture results were recorded during the subsequent clinical course at the time of each bronchoscopy.

After use of bronchial washings for clinical care purposes, any leftover material was processed for the research protocol and stored for mediator assays as described below. We classified patient samples and clinical data into early, which we defined as <72 hr. after injury, versus “later” time points, which we defined as any time point greater than 72 hours post injury and less than 14 days post injury. Levels of early mediators measured in bronchial washings were compared with the lowest PaO_2_/FiO_2_ ratio measured during the first 14 days post injury, as a quantitative marker of lung injury. PaO_2_/FiO_2_ less than 200 was a marker of more severe lung injury compared to PaO_2_/FiO_2_ ratios greater than 200 which indicated less severe lung injury. Study sample size was per convenience, and limited to consecutive eligible patients admitted and consenting to participate during a period of pilot funding.

Since the bronchoscopic samples used for the research would otherwise be discarded, the samples were collected and processed, but not stored in the repository until informed consent was obtained from the patient's legal authorized representative (LAR). Subjects who improved subsequently were given the opportunity to confirm or deny the LAR's informed consent. This study was approved by the UNC Biomedical Institutional Review Board.

### Clinical protocol for respiratory care of patients with suspected inhalation injury

All patients with suspected inhalation injury were automatically placed on the VDR-4® (Percussionaire Corporation, Sandpoint, ID) high-frequency percussive ventilator (HFPV) upon arrival to the burn center and stabilized on ventilator settings in accordance with low-tidal volume protective ventilation strategy.

All intubated patients with suspected inhalation injury underwent bronchoscopic evaluation within 24 hours of admission for the presence of inhalation injury. The bronchoscope was inserted into the endotracheal tube and the central airways inspected visually and graded for the presence of soot (minimum = 1, moderate = 2, severe = 3), mucosa inflammation (no = 0, yes = 1), mucosal sloughing (no = 0, yes = 1) and secretions (minimum = 1, moderate = 2, severe = 3). A total inhalation injury score (range 2–8) was calculated by adding the score for these four parameters at each bronchoscopy. After that, bronchial washings were performed in which 20 mL sterile saline lavage fluid was instilled and suctioned back from each mainstem bronchus, then collected in a suction trap. Bronchial washings were sent for culture as needed per clinical situation.

After the initial bronchoscopic evaluation, patients underwent subsequent bronchoscopies based on clinical necessity, *e.g.*, daily until all traces of visible soot were removed; as part of assessment of increased secretions or change in radiographic findings; before changing to another mode of ventilation; or as part of fever or elevated white blood cell evaluation. Ventilator management of these patients was done in collaboration between the surgical staff and respiratory therapists.

### Processing of bronchial washings and mediator assays

Bronchial washing specimens were transported on ice and processed within several hours of specimen collection. Briefly, samples were centrifuged at 300 g and aliquots of the resultant cell-free supernatant were stored at −80°C for subsequent mediator measurements. Neuropeptidase inhibitor was added to one aliquot from each specimen prior to freezing, for subsequent neuropeptide assays. A limited number of mediators was selected for assay in bronchial washings, as roughly representative of inflammatory (tumor necrosis factor alpha (TNF-α), interleukin-1 beta (IL-1β), IL-6, IL-8), immune (IL-10, IL-12p70, interferon gamma (IFNγ)), tissue damage and repair (double-stranded deoxyribonucleic acid (dsDNA), transforming growth factor beta-1 (TGF-β1)), and neuropeptide (substance P, calcitonin gene-related peptide (CGRP)) responses. Cytokines (IL-1β; IL-6; IL-8, IL-10; IL-12 p70; IFNγ; TNF-α) were measured using a multiplex ELISA platform (MesoScale Discovery, Gaithersburg, MD). TGF-β1 was measured using a commercial ELISA kit (R & D Systems, Minneapolis, MN). dsDNA was quantified using QuantIt™ PicoGreen® dsDNA Assay Kit (Invitrogen™ Ltd, Eugene, OR), a fluorescent nucleic acid stain. Substance P (R & D Systems, Minneapolis, MN) and CGRP (Peninsula Laboratories, San Carlos, CA) were measured using commercial ELISA kits. All assays were run according to manufacturer's instructions.

### Statistics

Subjects underwent 1–3 bronchoscopies during the initial 72 hr. post injury. To reduce the data to a single point per mediator and patient, if multiple samples were generated for a patient during the first 72 hr., the mediator data were averaged. The association between the average levels of mediators in bronchial washings collected in the first 72 hours, and the clinical outcome, as represented as the lowest PaO_2_/FiO_2_ ratio observed during the initial14 days post injury, was then evaluated using a multivariate regression model where confounding covariates like bacterial lower respiratory infection, visual severity of initial injury, gender, and ethnicity are included in the model. Specifically, we fitted the following regression model:
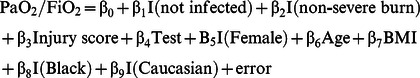
where *Test* was one of the measured mediators. All of the test variables were standardized so that they all had a mean of 0 and a standard deviation of 1.

## Results

*Patient Characteristics:* During the 8-month study period, 783 patients were admitted to the Burn Center for treatment of injuries. Of these, 64 were intubated and eligible for study under our protocol. Six patients were not approached for participation because their admission was too brief; 5 declined to participate; 3 were non-English speaking; and 7 died before consent could be obtained. Thus, 43 consented to participate. One enrolled patient was excluded from the data analysis due to lack of specimens obtained during the first 72 hours post-injury, and another 2 did not have sufficient material left for analysis, after clinical use of specimens. Thus, there were a total of 40 patients who comprise our study population.

Demographic data for the 40 patients studied are shown in Table 1. Cutaneous burn size ranged from 0–85% total body surface area (TBSA), with a mean of 18% TBSA. The plurality of patients suffered their injuries due to flame injuries (40%), followed by house fires (30%) and explosions (25%). Our study population demographics were similar to those published trends in the 2011 Summary Report of the National Burn Repository, American Burn Association (http://128.121.65.211/2011NBRAnnualReport.pdf). All demographic parameters were similar between the subgroups who had PaO_2_/FiO_2_ <200, and PaO_2_/FiO_2_ ≥200.

**Table 1:** Demographic and clinical characteristics of study population.

	PaO_2_/FiO_2_ >200	PaO_2_/FiO_2_ <200	Total
N	24	16	40
Age (yrs)	46.4±4.0	44.4±4.9	45.6±3.1
Gender (% female)	5/24 (21%)	4/16 (25%)	9/40 (23%)
BMI	27.9±1.7	28.3±2.3	28.1±1.3
% TBSA	17.7±3.6	18.6±4.3	18.3±2.9
Bronchoscopies*	2.6±0.3	3.8±0.6	3.1±0.3
Abnormal chest radiograph**	19/24 (79%)	15/16 (94%)	34/40 (85%)
Days on ventilator	23.6±5.0	34.1±8.0	27.8±4.4
Days in hospital	31.5±4.8	50.9±7.2	39.3±4.3
Mortality	4/24 (17%)	2/16 (13%)	6/40 (15%)

* Within 1^st^ 14 days after injury.

** Read by radiologist as having either infiltrates, edema, or atelectasis.

After injury, length of hospital stay ranged from 2–136 days, (mean 39 days). The number of days on the ventilator was variable and averaged 28 days; this time was determined not only by the severity of inhalation injury or pulmonary status, but also by % TBSA and co-morbid conditions. Mortality was 15% for the study population. Mean %TBSA burns, number of days in hospital, and number of days on ventilator were all slightly higher in the subgroups who had PaO_2_/FiO_2_ <200, vs. PaO_2_/FiO_2_ ≥200. Percent mortality was slightly lower in the PaO_2_/FiO_2_ <200 subgroup (Table 1).

**Figure 1:**
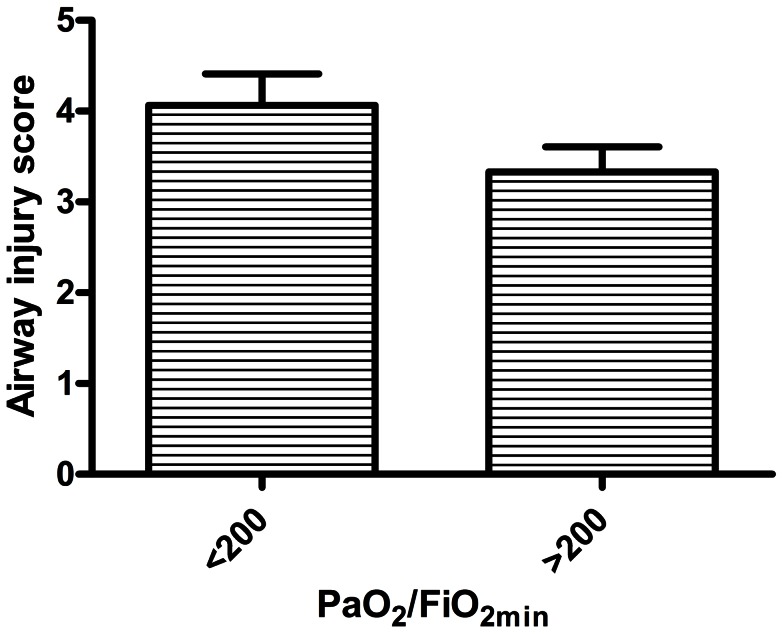
Comparison of airway injury score (graded for soot, inflammation, epithelial sloughing and secretions at <72 hr post injury), between patients with the lowest PaO_2_/FiO_2_ ratios during the first 2 weeks post injury (PaO_2_/FiO_2_) of <200 (N = 16), and patients with PaO_2_/FiO_2_ ≥200 (N = 24). Bars represent mean ± SE. P = 0.11, t-test with Welch's correction for unequal variances.

**Figure 2:**
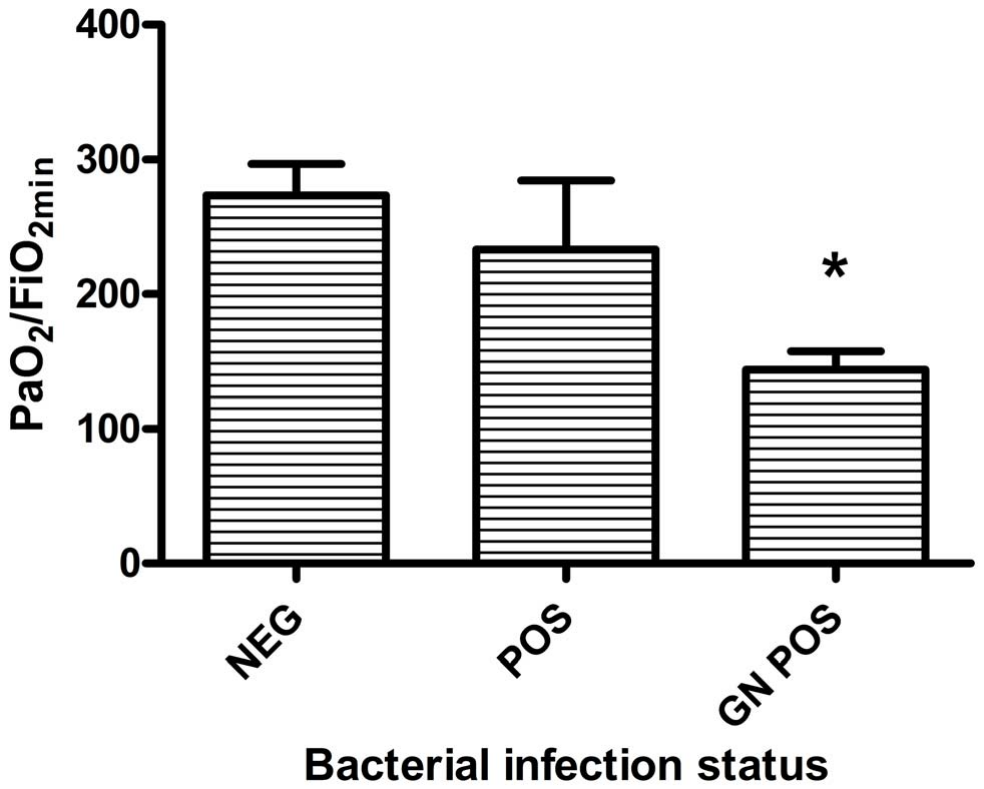
Comparison of the lowest PaO_2_/FiO_2_ ratios during the first 2 weeks post injury (PaO_2_/FiO_2_) among patients with no bacterial pathogens isolated from respiratory secretions during the first 2 weeks (NEG), patients with bacterial pathogens of any type (POS), and patients with gram negative bacteria (GN POS). Patients in whom no bacterial cultures were collected are not included in this analysis. * = P<.05 vs. NEG.

While mean % neutrophils in bronchial washings during the first 72 hr. post injury was higher in the group with PaO_2_/FiO_2_ <200 (84.5±3.6%) than in the group with PaO_2_/FiO_2_ >200 (78.7±3.2%), the difference was not statistically significant (P = 0.23). In initial univariate analyses, % TBSA burns did not appear to predict PaO_2_/FiO_2_. Initial airway injury score via bronchoscopic grading was on average higher in the group with PaO_2_/FiO_2_ <200, but this difference was not statistically significant (P = 0.11; Figure 1). While having any bacterial respiratory infection (defined as at least one positive lower respiratory culture for bacterial pathogens within the first 14 days post injury) was associated with only slightly lower PaO_2_/FiO_2_ than if there was no infection (233±52 vs. 273±24), the subgroup in which a gram negative bacterial pathogen was isolated had a mean PaO_2_/FiO_2_ of only 144±14 (P<0.001 vs. no infection). (Fig. 2). These parameters were included as covariates in the multivariate model. The bacterial organisms that were identified from BALF in the first 14 days after injury include: gram-positive organisms, e.g., Oxacillin resistant *Staphylococcus aureus* and gram negative organisms, e.g., *Haemophilus influenza* and *Klebsiella pneumonia* (See Table 2). This study was not designed to determine an association between inflammatory mediators, lung injury and infection, but will be explored in future studies.

**Table 2:** Microbiologic data of positive respiratory samples collected from subjects in the first 14 days according to gram stain.

Gram positive organisms	Gram negative organisms
*ORSA, OSSA, Streptococcal pneumoniae, diptheroids*	*Haemophilus influenzae, Enterobacter aerogenes, Moraxella catarrhalis, Acinetobacter baumannii, klebsiella pneumoniae, Escherichia coli*

### Relationship of soluble mediators in bronchial washings to ALI

Table 3 shows levels of the mediators we measured in bronchial secretions, in patients with lower PaO_2_/FiO_2_ vs. higher PaO_2_/FiO_2._ In the multivariate regression model, each measured soluble factor in early mainstem bronchial washings was tested as a predictor of PaO_2_/FiO_2_. Bacterial respiratory infection during the first 14 days, % TBSA burn, initial airway injury score, age, gender and race were treated as potential confounders in the model.

**Table 3:** Mediator concentrations in mainstem bronchial washings, averaged for samples obtained during the first 72 hr post injury.

	PaO_2_/FiO_2_ >200	PaO_2_/FiO_2_ <200
N	24	16
IL-1β (pg/ml)	1175±516	3091±1761
IL-6 (pg/ml)	1747±945	2389±797
IL-8 (pg/ml)	23,515±9277	27,393±7737
IL-10 (pg/ml)	35.2±4.7	87.1±18.6
IL-12p70 (pg/ml)	20.1±2.2	16.5±2.8
TNF-α (pg/ml)	229.2±70.5	1046±310.6
TGF-β1 (pg/ml)	245.3±60.0	371.3±157.6
IFNγ (pg/ml)	81.2±11.3	102.1±20.5
CGRP (ng/ml)	0.4±0.1	4.5±3.6
dsDNA (ng/ml)	2485±619	6049±1416

Data are shown as mean ± SE.

Substance P was undetectable in most specimens. IL-1β, IL-6, IL-8, TNF-α, and CGRP were readily measureable but were not significant predictors of PaO_2_/FiO_2_. IFNγ (P = 0.051), TGF-β1 (P = 0.10), and dsDNA (P = 0.11) were also not statistically significant predictors of PaO_2_/FiO_2_ though their P values in the multivariate model were closer to the predetermined level of significance. For descriptive purposes, the mean ± SE data for these mediators are shown in Table 2, for the patient groups with PaO_2_/FiO_2_ <200 vs. PaO_2_/FiO_2_ ≥200.

**Figure 3:**
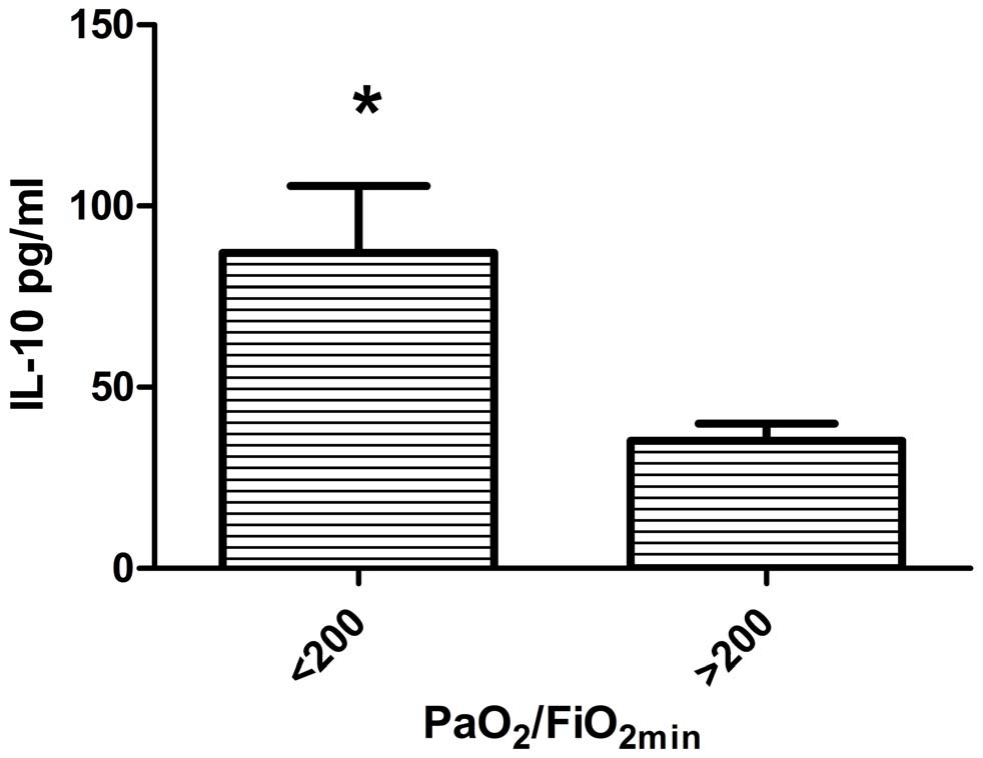
Comparison of averaged early (<72 hr post injury) IL-10 concentrations in mainstem bronchial washings, between the lowest PaO_2_/FiO_2_ ratios ratios during the first 2 weeks post injury (PaO_2_/FiO_2_) of <200 (N = 16), and patients with PaO_2_/FiO_2_ ≥200 (N = 24). Bars represent mean ± SE. * P<0.05, t-test with Welch's correction for unequal variances.

**Figure 4:**
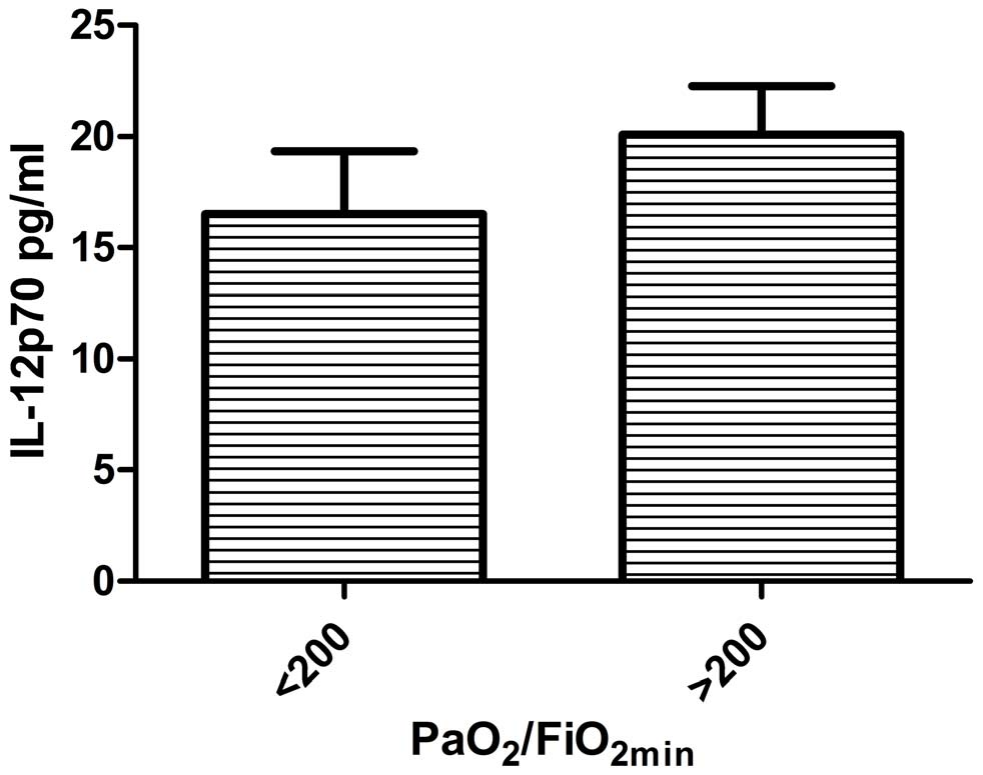
Comparison of averaged early (<72 hr post injury) IL-12p70 concentrations in mainstem bronchial washings, between the lowest PaO_2_/FiO_2_ ratios during the first 2 weeks post injury (PaO_2_/FiO_2_) of <200 (N = 16), and patients with PaO_2_/FiO_2_ ≥200 (N = 24). Bars represent mean ± SE.

**Figure 5:**
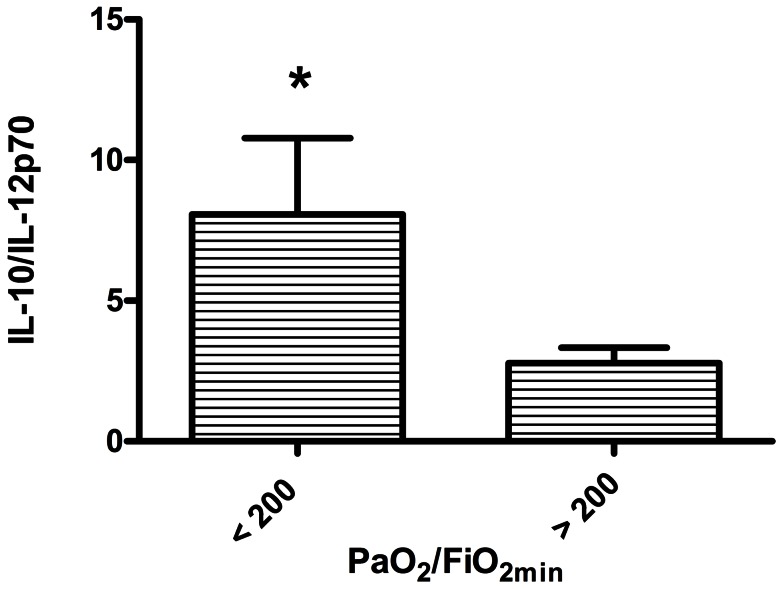
Comparison of IL-10 to IL-12p70 ratios in mainstem bronchial washings, between patients with the lowest PaO_2_/FiO_2_ ratios during the first 2 weeks post injury (PaO_2_/FiO_2_) of <200 (N = 16), and patients with PaO_2_/FiO_2_ ≥200 (N = 24). Bars represent mean ± SE. * P<0.05, t-test with Welch's correction for unequal variances.

IL-12p70 and IL-10 were significant (P<.05) predictors of PaO_2_/FiO_2_. For descriptive purposes these factors are shown in relation to the lowest PaO_2_/FiO_2_ (>200, indicative of less severe lung injury versus <200, indicative of more severe lung injury) observed in the first 14 days after injury in Figures 3 and 4. For IL-12p70, higher concentration was associated with greater PaO_2_/FiO_2_ ratios, indicating less severe lung injury. Greater IL-10 levels were associated with lower PaO_2_/FiO_2_ ratios or more severe lung injury. Because dilution of native airway secretions by lavage fluid was not accounted for in the preceding analyses, we also calculated the ratio of early IL-10 to early IL-12p70 for each patient and compared the PaO_2_/FiO_2_ <200 subgroup to the PaO_2_/FiO_2_ >200 subgroup. The IL-10/IL-12p70 ratio was significantly higher for the PaO_2_/FiO_2_ <200 subgroup (8.1±2.7, vs. 2.8±0.5; P = 0.028; Figure 5).

## Discussion

In a prospectively studied cohort of endotracheally intubated or tracheotomized inpatients with burn and inhalational injuries, we observed that early cytokine patterns in airway secretions may be predictive of the severity of lung injury. In particular, elevated IL-10 and decreased IL-12p70 may be associated with progression to more severe lung injury, even after accounting for the potential confounders of burn severity, bacterial lower respiratory infection, and the severity of initial injury in the large central airways. Elevated levels of IFNγ, TGF-β1, and dsDNA also tended toward association with more severe lung injury, but were not statistically significant in our multivariate model.

For many years, the immune response to serious injury has been defined as an initial pro-inflammatory response that is quickly followed by a systemic inflammatory response syndrome (SIRS), which if uncontrolled, results in early multiple organ dysfunction syndrome (MODS) and, ultimately death In most patients however, after a period of relative stability, a compensatory anti-inflammatory response syndrome (CARS) develops with associated immunosuppression and increased risk of infection that if uncontrolled, can also result in MODS and death. The controlling mechanisms for initiating and sustaining the development of SIRS and CARS have not been fully elucidated and attempts to modulate either response with cytokine therapy have largely been unsuccessful [12], except in very specific and controlled animal models of burn injury and other models of trauma [13]–[19]. The failure of this intervention may be because the immune phenotype after injury has not been fully defined, especially late after injury when immune failure and subsequent infection, sepsis, MODS and ALI are most likely. We, and others, have attempted to define the cytokine response in mouse models and humans and it is clear that SIRS to CARS progression is overly simplistic, with dynamic alterations in specific cell populations and specific cytokine responses at different physiologic sites. We argue that the human lung represents a crucial site to investigate the immune response as a common cause of death after trauma is bacterial pneumonia. Investigation of perturbations of the IL-10/IL-12 axis provides the foundation for further studies to define the cellular and molecular mechanisms. These studies will lead to specific immunotherapies that will reduce length of stay and improve survival after inhalation injury.

Ours is one of several recent studies to describe mediator concentrations in bronchial washings in burn/inhalational injury patients. Kurzius-Spencer et al. [20] measured cytokines in serial tracheal aspirates during the first 72 hr. post injury in a smaller series of intubated smoke inhalation victims, and reported a direct relationship between IL-8 levels and reduction of risk for ARDS, but no temporal changes in IL-10 or relation to lung injury risk during this early time period. Davis *et al*. [21] found that mortality was associated with immune hyporesponsiveness manifested by generally lower cytokine levels of immunomodulators, e.g., IL-1β, IL-8, and IL-10, in BALF obtained within the first14 hrs after injury, and decreased release of cytokines by BALF leukocytes in short-term culture *ex vivo*. Our measured cytokine concentrations were higher (Table 2) than those reported by Davis *et al*., probably due to less dilution of airway secretions in our bronchial washings, compared to BALF. In an separate report from the same cohort, elevated cytokine levels were associated with more severe bronchoscopic grades of inhalation injury, with the exception of IL-12, which was reduced compared to control subjects [22], which are consistent with our findings that showed lower levels of IL-12p70 in association with more severe lung injury.

Despite many studies, there is still a lack of reliable prognostic markers of ALI. Some of the inflammatory mediators that have been examined with respect to ALI include: IL-1β, TNF-α, IL-8, ICAM-8, plasminogen activator inhibitor-1, Protein C, IL-10/TNF-α ratio, and IL-6. Serum and BALF levels of IL-1β have been observed to strongly correlate with poor outcome and severity of lung injury [19], but require careful study due to the complexity of the IL-1β inhibitory system also present in these fluids. Recent studies by others have demonstrated IL-1β and other inflammasome-mediated cytokines may serve as a novel biomarker for ALI [23]. This requires further study, as we have shown that inflammasome related genes are rapidly down regulated after burn injury [24]. TNF-α has also been studied and correlates well with severity of ALI, but is complicated to measure and correlate with ALI as increased inhibitory soluble TNF-α receptors also need to be measured in parallel. IL-6 is produced in response to inflammation and has been implicated in the progression to ALI and cardiovascular disease [25]. Even with these studies, a specific subset of these indices in combination has been suggested as having the best predictive value [26]. IL-8 and SP-D are the best performing combination when predicting clinical outcome. Interestingly, this study did not include IL-10 within their tested group of biologic markers. Increased levels of lung IL-10 have been described in experimental animal models for acute lung injury due to lung contusion and aspiration [27], and alcohol plus burn exposure [28]. Elevated plasma IL-10 and several other mediators were recently reported to be markers for progression to ALI in patients after traumatic injuries [29]. Relative quantities of cytokines present in the airways early after injury may help direct immune and inflammatory responses toward an injurious rather than a reparative response, perhaps via effects on macrophage differentiation and downstream cytokine patterns [30]. Wu et al. [31] reported increased endogenous IL-10 levels in the lungs of mice after LPS-induced acute lung injury. Administration of exogenous IL-10 appeared to have a protective effect. This suggests that IL-10 may be beneficial, rather than deleterious, with respect to acute lung injury, under some circumstances. A recent report of markedly suppressed cytokine secretion by splenocytes recovered at autopsy from patients dying of sepsis, compared to patients recovering, supports the importance of cytokine patterns in predicting clinical outcomes of critically ill patients [32].

While it did not reach statistical significance, we also observed elevated DNA concentrations in bronchial washings, on average, in patients with lower PaO_2_/FiO_2_ ratios. Potential sources of DNA in airway secretions include directly damaged epithelial cells and inflammatory cells (e.g., neutrophils) responding to injury [2], [33], [34]. Our data cannot differentiate among these cellular sources, since all the factors that we measured could be derived from multiple cell types. Whatever its source, it is possible that DNA may function as an endogenous damage-associated molecular pattern (DAMP) after inhalational injury, for example driving the inflammatory component of ALI via interactions with TLR9 on neutrophils [35], [36].

Experimental animal model data and clinical experience have linked bacterial infection to poor outcomes in burn/inhalational injury, particularly with gram-negative pathogens such as *Pseudomonas* [37], [38]. Our data also suggest a potential relationship between gram-negative bacteria in the respiratory tract and ALI (Fig. 2). However, our observational study design in which cultures were not uniformly or systematically collected does not allow strong conclusions about this relationship. While associations between bacterial infection and risk for ALI in the burn/inhalational injury setting are not surprising, our data justify inclusion of infection as a potential confounder in the multivariate model exploring the roles of specific host defense factors in development of ALI.

Our study has several limitations. Since we relied on clinically indicated procedures and samples in a critically ill population, both our patient population and our sample collection frequency were variable. Therapy was individualized as well, which means that there were multiple interventions (e.g., fluid resuscitation, antibiotics) occurring which could have affected the data we measured. Even under the strictest of protocols, studies involving the analysis of bronchial lavages have the potential to introduce variability in sample collection due to the dilution of native secretions with exogenous lavage fluid and incomplete retrieval of infused lavage fluid. This variability is difficult to accurately account for amongst patient samples. In this regard, the use of cytokine ratios (Fig. 5), while biologically artificial, may be a more reliable indicator of data comparisons between groups, since dilution factors drop out of the analysis.

For our analysis, we used the lowest PaO_2_/FiO_2_ ratio at any time during the first 14 days after injury, as our primary outcome of lung injury, and we presented our data for descriptive purposes comparing patients with a ratio of <200, indicating more severe lung injury, to those with ratios >200, indicating less severe lung injury. The majority of our patients also had radiographic abnormalities (Table 1), however, the relationship of these abnormalities to the degree of severity of lung injury make interpretation problematic. Thus, we did not attempt to sort patients based on the complete clinical definition of ARDS [39], [40], which also includes chest x-ray and left atrial pressure parameters.

Despite these limitations, we believe this study represents a novel and useful approach for translational research into pulmonary complications after inhalational injury. While our data do not suggest specific treatments, understanding this biology is essential for developing interventions to minimize respiratory tract morbidity and mortality in these patients. Our observations support testable hypotheses regarding the pathophysiology of ALI and gram-negative infection following inhalation of soot by persons injured in fires. For example, a hypothesis consistent with our results is that initial airway injury increases levels of airway IL-10, which if prolonged, results in immune suppression and increased risk for gram-negative infection. In parallel, dsDNA in the airways may function as a damage-associated molecular pattern (DAMP), mediating early innate immune activation that contributes to development of lung injury. Persistent exposure to this DAMP could contribute to suppression of TLR and inflammasome dependent innate immune processes, also leading to increased risk of gram-negative infection. Mechanistic studies using experimental models will be required to test these hypotheses. Additionally, continued investigation of factors that modify airway cell responses to soot will likely identify other mechanisms which contribute to both the severity of lung injury and risk of airway infection.
